# Supplementary material for: Complexome profiling of the Chlamydomonas psb28 mutant reveals TEF5 as an early PSII assembly factor
Source: Plant Cell. 2025 Mar 17;37(6):koaf055. doi: 10.1093/plcell/koaf055 (PMC12164590; doi:10.1093/plcell/koaf055)
Supplement: koaf055_Supplementary_Data [file koaf055_supplementary_data.zip › psb28_interactive.zip!/plots/intensity/qualityPlots/peptides/_Cre07.g321300.t1.2.html]

### Cre07.g321300.t1.2

Trivial name:   
SeqCoverage [median]: 16.70  
Mass: 21.70 kDa  
  
 

| Sequence | MissedCleavage | Mass [Da] | wtIntensity | muIntensity | Score |
| --- | --- | --- | --- | --- | --- |
| DGSRLEITGIEK | 1 | 1316.69 | 0.00 | 535.27 | 54.70 |
| FKEIADHINR | 1 | 1241.65 | 0.00 | 598.11 | 44.61 |
| LVITNTSPLFK | 0 | 1231.72 | 0.00 | 2259.02 | 91.85 |
|  |  |  | 0.0 | 3392.4 |  |
